# One million dog vaccinations recorded on mHealth innovation used to direct teams in numerous rabies control campaigns

**DOI:** 10.1371/journal.pone.0200942

**Published:** 2018-07-26

**Authors:** Andrew D. Gibson, Stella Mazeri, Frederic Lohr, Dagmar Mayer, Jordana L. Burdon Bailey, Ryan M. Wallace, Ian G. Handel, Kate Shervell, Barend M.deC. Bronsvoort, Richard J. Mellanby, Luke Gamble

**Affiliations:** 1 Mission Rabies, Cranborne, Dorset, United Kingdom; 2 The Roslin Institute and The Royal (Dick) School of Veterinary Studies, Division of Genetics and Genomics, The University of Edinburgh, Hospital for Small Animals, Easter Bush Veterinary Centre, Roslin, Midlothian, United Kingdom; 3 Worldwide Veterinary Service, Cranborne, Dorset, United Kingdom; 4 Poxvirus and Rabies Branch, Centers for Disease Control and Prevention, Atlanta, Georgia, United States of America; 5 The Royal (Dick) School of Veterinary Studies, Division of Veterinary Clinical Studies, The University of Edinburgh, Hospital for Small Animals, Easter Bush Veterinary Centre, Roslin, Midlothian, United Kingdom; Wistar Institute, UNITED STATES

## Abstract

**Background:**

Canine transmitted rabies kills an estimated 59,000 people annually, despite proven methods for elimination through mass dog vaccination. Challenges in directing and monitoring numerous remote vaccination teams across large geographic areas remain a significant barrier to the up-scaling of focal vaccination programmes to sub-national and national level. Smartphone technology (mHealth) is increasingly being used to enhance the coordination and efficiency of public health initiatives in developing countries, however examples of successful scaling beyond pilot implementation are rare. This study describes a smartphone app and website platform, “Mission Rabies App”, used to co-ordinate rabies control activities at project sites in four continents to vaccinate over one million dogs.

**Methods:**

Mission Rabies App made it possible to not only gather relevant campaign data from the field, but also to direct vaccination teams systematically in near real-time. The display of user-allocated boundaries on Google maps within data collection forms enabled a project manager to define each team’s region of work, assess their output and assign subsequent areas to progressively vaccinate across a geographic area. This ability to monitor work and react to a rapidly changing situation has the potential to improve efficiency and coverage achieved, compared to regular project management structures, as well as enhancing capacity for data review and analysis from remote areas. The ability to plot the location of every vaccine administered facilitated engagement with stakeholders through transparent reporting, and has the potential to motivate politicians to support such activities.

**Results:**

Since the system launched in September 2014, over 1.5 million data entries have been made to record dog vaccinations, rabies education classes and field surveys in 16 countries. Use of the system has increased year-on-year with adoption for mass dog vaccination campaigns at the India state level in Goa and national level in Haiti.

**Conclusions:**

Innovative approaches to rapidly scale mass dog vaccination programmes in a sustained and systematic fashion are urgently needed to achieve the WHO, OIE and FAO goal to eliminate canine-transmitted human deaths by 2030. The Mission Rabies App is an mHealth innovation which greatly reduces the logistical and managerial barriers to implementing large scale rabies control activities. Free access to the platform aims to support pilot campaigns to better structure and report on proof-of-concept initiatives, clearly presenting outcomes and opportunities for expansion. The functionalities of the Mission Rabies App may also be beneficial to other infectious disease interventions.

## Introduction

An estimated 59,000 people die from rabies annually, despite decades of evidence demonstrating that mass dog vaccination can effectively eliminate the disease in both people and dogs [1–4]. To achieve the goal of dog-mediated rabies elimination by 2030 set by WHO, OIE, FAO and other global experts, there is a requirement to expand mass dog vaccination efforts in many endemic countries which have yet to take the first steps towards national programmes [5]. Although vaccination of at least 70% of the dog population has been deemed practically feasible in many areas [6,7], further work is needed to overcome remaining political, financial and operational barriers to implementing successful national efforts [4,5].

It is estimated that an additional 248 million dogs need to be vaccinated globally every year to achieve the 2030 elimination target [5]. The development of tools and approaches to improve the efficiency, monitoring and evaluation of large scale mass dog vaccination initiatives has the potential to greatly enhance the impact of finite resources in addition to identifying areas where a change in approach is needed. Successful elimination of the rabies virus through mass dog vaccination requires synchrony of vaccination activities achieving homogenous coverage across large geographic areas [8–10]. At the operational level this involves the direction of teams to vaccinate over 70% of the dog population in each area over a short period of time before moving to the next area. Focal direction of teams is challenging where areas are not demarcated by easily distinguishable boundaries, increasing the likelihood of duplicated effort in some areas whilst not reaching others entirely.

Innovation in mobile health (mHealth) solutions have enhanced disease control activities in resource-limited settings by improving the speed of data capture, management and analysis [11–15]. Specific examples include mass polio and cholera vaccination and, more recently, for gathering rabies surveillance data [16–18], however methods for its application to mass dog vaccination have not yet been reported. mHealth has the potential to improve management and geographic coordination of large scale rabies control efforts from the day-to-day direction of mass dog vaccination, public education and surveillance teams at the community level, whilst also enabling rapid assimilation and review of national activity.

This study describes the incorporation of a novel smartphone app and website platform, the “Mission Rabies App” into rabies control activities in sixteen countries across Africa, Asia, Latin America and Europe to not only gather field data pertinent to monitoring project output and impact, but also to direct teams to specific geographic areas and coordinate education and vaccination activities.

## Methods

### Ethics statement

Ethics approval for analysis of data relating to mass dog vaccination was obtained from University of Edinburgh’s Veterinary Ethics Research Committee (64/5: Investigation of rabies vaccination approaches). All data were collected as part of rabies control public health campaigns, with permission from the relevant local authorities in the areas of work. All data were stored on secure cloud-based servers with restricted access to authorised project management staff.

### Study area and project design

Vaccination campaigns were implemented by the international Non-Governmental Organisation (NGO) Mission Rabies in partnership with local municipalities, governments and NGOs in Blantyre/Chiradzulu/Zomba Districts (Malawi), Goa State (India) and Ranchi City (India). In these core project sites, the Mission Rabies App was used throughout the year to support mass dog vaccination, school education and emergency community response to reported rabies cases. Additional proof-of-concept mass dog vaccination campaigns, vaccinating approximately 5,000 dogs, were conducted for two week periods annually in Negombo (Sri Lanka), Meru (Tanzania) and Koch-Goma (Uganda) from 2015 without education or rabies response components. In 2017, the system was adopted in Haiti through partnership with Poxvirus and Rabies Branch of Centre for Disease Control and Prevention (CDC), and Ministere de L’Agriculture des ressources Naturelles et du Developpement Rural (MARNDR) for coordination of the national dog vaccination campaign. Independent partner organisations also used the Mission Rabies App for purposes of coordinating dog sterilization work, dog enumeration studies and mass dog vaccination in Goa (India), Kabul (Afghanistan), Kragujevac (Serbia), Sarajevo (Bosnia and Herzegovina), Baku (Azerbaijan), Yerevan (Armenia), Praia de Faro (Portugal). In a number of project sites partner organisations complement rabies control through interventions to improve dog population management. Dogs undergoing surgery routinely received rabies vaccine at the same time in all projects entering data in the Mission Rabies App.

The structure of mass dog vaccination campaigns varied depending on local dog demographics and ownership practices; in Malawi, Uganda and Tanzania a combination of central point (CP) and door-to-door (DD) vaccination was used, whilst in India and Sri Lanka teams moved through the streets vaccinating dogs using DD and catch-vaccinate-release (CVR) to access dogs [19,20]. Vaccination team size varied depending on approach with CP/DD teams generally consisting of two to three people, whilst CVR teams were usually seven people. Dogs were parenterally vaccinated with Nobivac Rabies (MSD Animal Health) and temporarily marked with non-toxic paint on the forehead to enable assessment of coverage. Direct dog sight surveys and household questionnaires were conducted in the days following vaccination of a region to assess vaccination coverage [19,20].

Education work in core project sites involved trained education officers delivering rabies education classes in schools throughout the regions of work. Education teams consisted of one or two people following a set schedule to visit regions before the arrival of vaccination teams, increasing awareness of the upcoming vaccination activities. Classes were delivered in the local language and lasted 15–30 minutes covering topics of bite avoidance, wound washing and post exposure prophylaxis as well as aspects of responsible dog ownership and dog population management relevant to the local setting.

Emergency rabies response services were established in core project sites to respond to suspected canine rabies cases reported by the community. These serve to prevent ongoing transmission of rabies virus to humans and other animals by removing the suspected animal, as well as monitoring the impact of vaccination efforts and improving animal welfare through humane management of rabid animals. Emergency canine rabies response teams comprised of at least two dog catchers, a driver and a veterinarian.

### Equipment and data security

Development of the Mission Rabies App began in April 2014 and was launched on field projects in November 2014. Improvements and expansion in functionality and design have taken place in response to field experience and user feedback to meet local project needs. Access to the basic functionalities of the system were made available free-of-charge from October 2017. Projects for external users were created upon application and available for download in android or iOS versions from the google Play Store and Apple App Store respectively.

Since launch, data were collected using a wide variety of smartphone devices, including Samsung Galaxy J1 and Samsung Galaxy J2, running the Android 4.4.4 (KitKat) operating system and above on 4.3 inch screen displays. Portable power banks were used to charge phones where necessary in the field. The backend database was managed using Microsoft SQL Server and the interface was written in C# and hosted on asp.net webform. The Android application was written in JAVA and the iOS application was written in Objective C.

**Fig 1:**
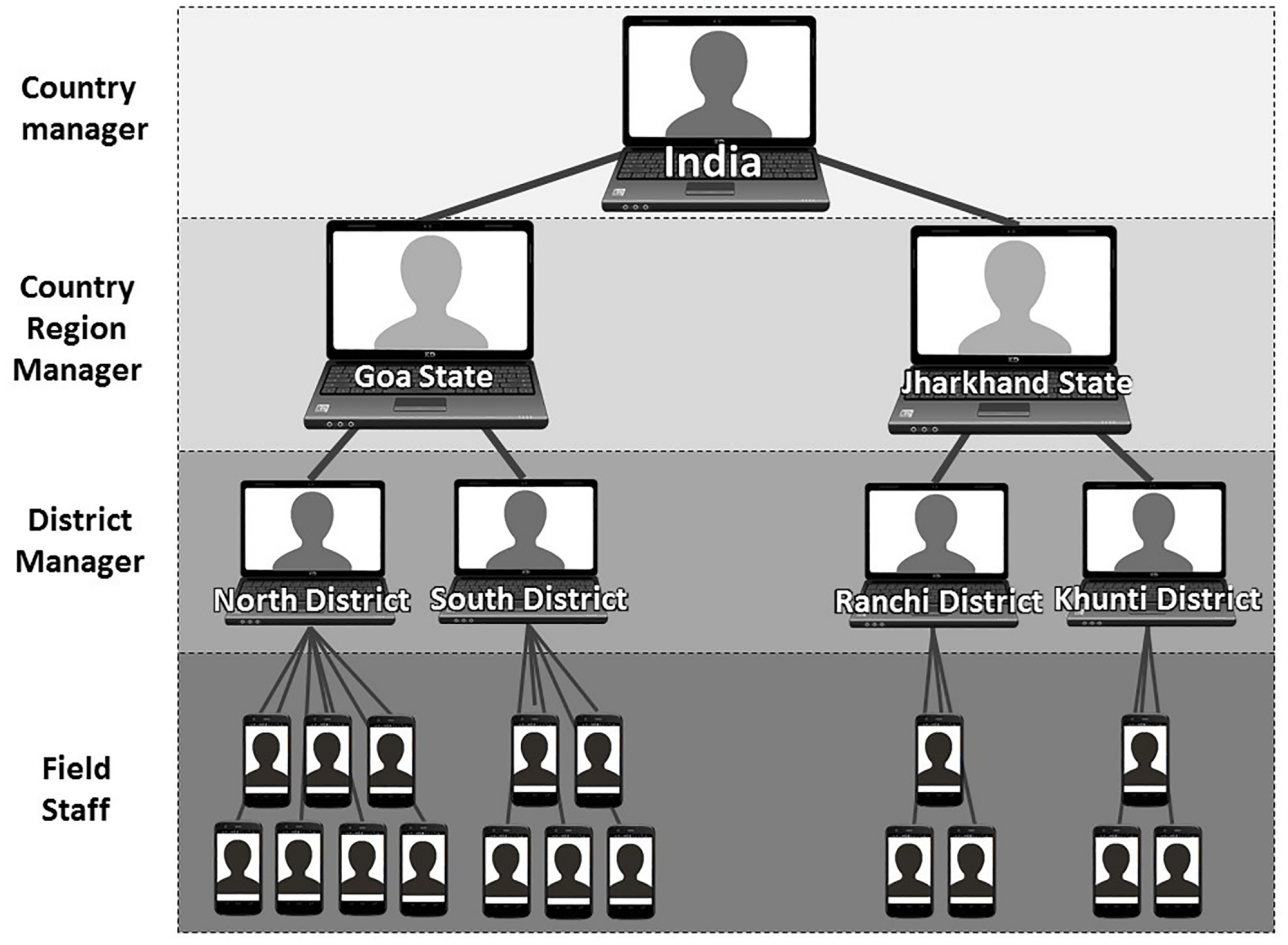
Illustration of user access structure to data within the Mission Rabies App at different levels within existing projects in India. Icons indicate how users interact with the system, by laptop or smartphone.

Data entered on secure smartphones were stored offline locally to the handset before being uploaded through SSL certificate secured connections to a cloud based server, which were accessed through a password protected website. Partition of data by project enabled restriction of access by individual users only to data that is relevant to their field of work. Fig 1 shows an illustration of the structure of data access within existing projects in India, however the same model was applied in other countries with flow of data and restriction of access at the user, district, region and country levels.

### Teams and training

The Mission Rabies App was used by staff to record information about operational outputs in each area of work; 1) mass dog vaccination 2) dog population surveys 3) education in schools and 4) emergency canine rabies response. The app was used by trained local staff in all project sites, with varied previous experience using smartphones and educational level, however the majority of users were educated to high-school level. Each project site had a project manager who was responsible for monitoring operational output and coordinating geographic direction of vaccination, survey and education teams. For the most part, these staff were non-native to the country of work, however local project managers successfully implemented the system in India. Project managers had prior abilities in basic use of computers, Microsoft Office tools and online systems and received approximately one hour of initial training in use of the web platform, followed by additional remote training through streamed online videos about updates to the system.

Training varied by project location and role, however training in data entry using the app generally took place over half a day, including interactive assessment and data review. Refresher training was conducted annually and when new staff were recruited, with debrief feedback sessions at the end of mass campaigns. Periodic checks through co-supervision by management staff in the field during mass vaccination campaigns were used to correct any variation from standard operating procedures. Further information about the training protocol for education officers and retention of rabies understanding was described by Bailey et al (2018) [21].

### Data collection

The initial purpose of the app system was to gather data from the field for central collation and review by project management staff in near real-time, before functionalities were expanded to enable geographic team direction.

**Fig 2:**
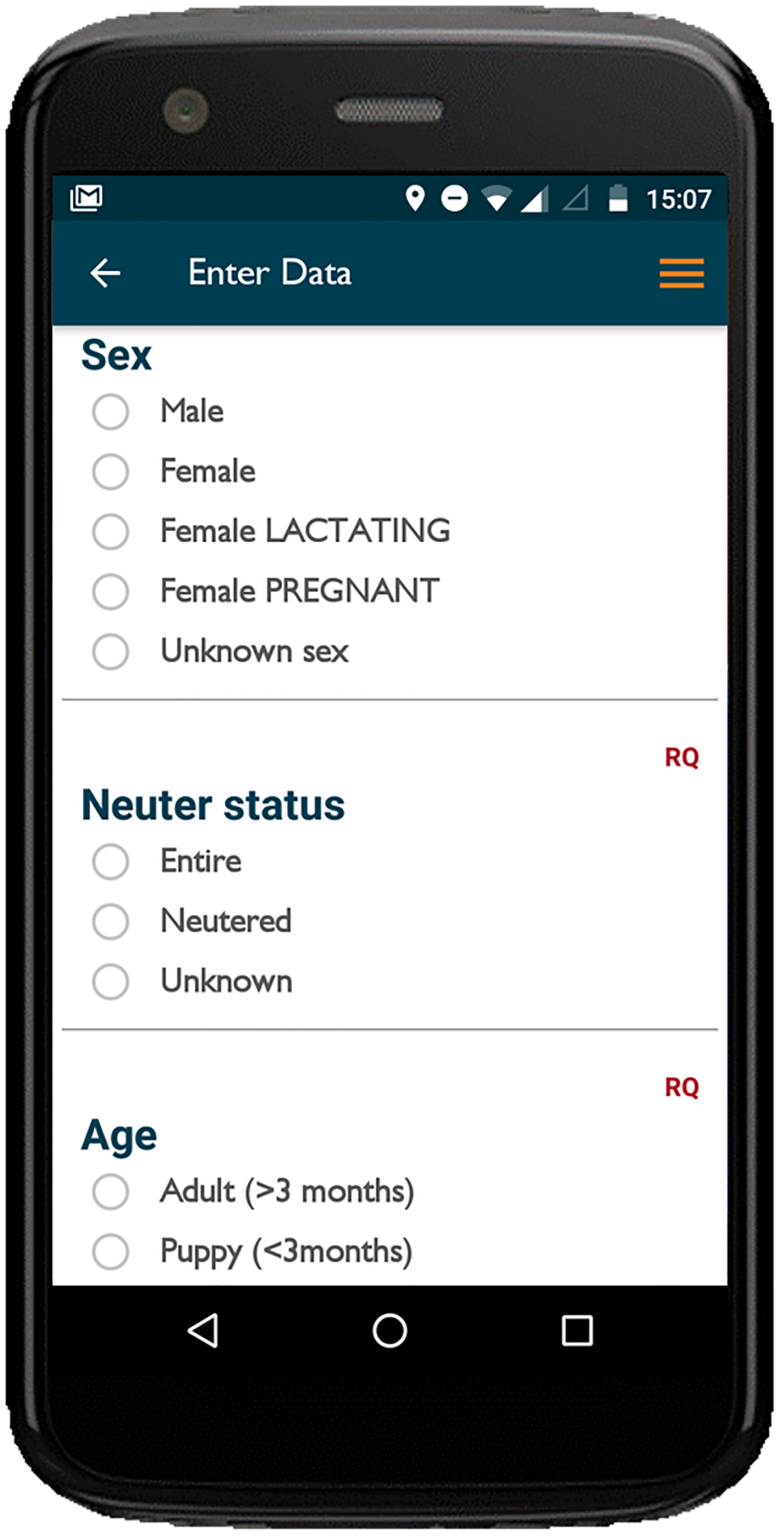
Example of data entry form as displayed on the smartphone handset. ‘RQ’ indicates a required field making entry of data compulsory before form completion.

Data were entered on customised forms, which were created on the web-interface and assigned to individual projects. Forms were grouped by category according to the type of data being entered; vaccination, survey, education, rabies response (S1 Table). Dynamic question logic formats with branching and dependent question logic enabled more complex form structures and compulsory fields ensured completeness of data. Questions were displayed in groups on scrolling screens to facilitate rapid data entry and, where necessary, division of long surveys into workable chunks (Fig 2).

During initial pilot campaigns, more comprehensive data about each dog were collected to fulfil grant requirements and to generate an understanding of population size, structure and ownership for the benefit of planning future work. In most cases an additional staff member was employed in each vaccination team to record data about the dogs being vaccinated, however for implementation in the Haiti National campaign, efficiency was improved beyond the initial pilot vaccination phase by only recording basic information about each dog (date, time, GPS location) which could be captured with a single button in the app, without entry of dog-specific data, therefore negating the need for additional staff.

The app periodically connected to the server to check for changes to forms and map boundaries, after which app functionalities could be used offline in the field. Users uploaded (synchronised) form data from phones when data connection was available, typically at the end of each day, usually via a Wi-Fi connection at the campaign office, or via 3G when returning to regions with good connection. Connection was generally poor in rural areas, however vaccine distribution points were often located in urban centres where connection is more available, allowing for data upload at least once a day. The data were automatically compiled into a web-based database for access by the project manager.

### Team direction

**Fig 3:**
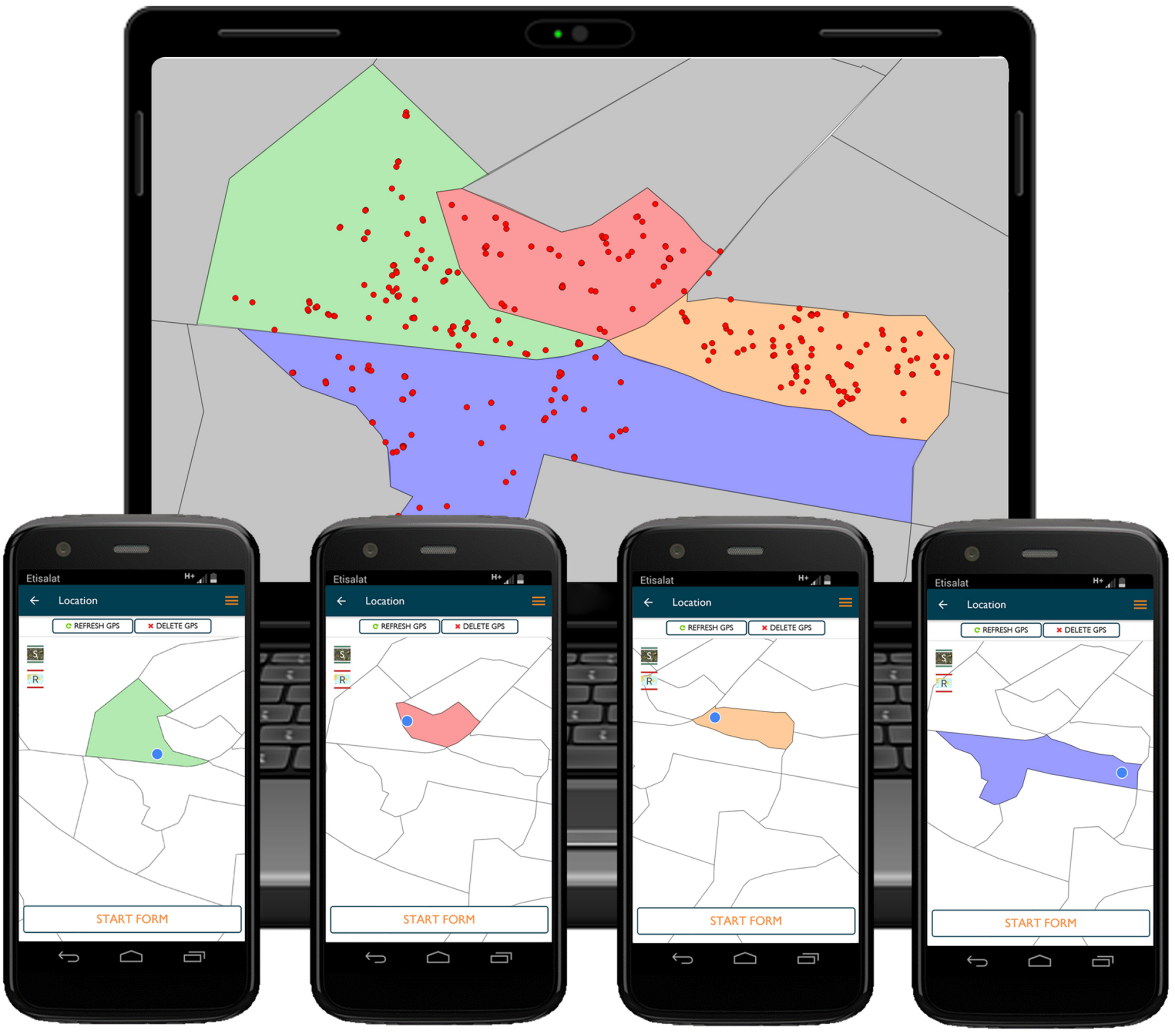
Illustration of maps and data as viewed by the project manager on the web platform and smartphone handsets. The project manager assigns coloured regions to vaccination teams in the web platform, shown here in the computer illustration. These regions are then displayed on each team’s smartphone and used to intensively work within that region (smartphone inserts). Once the region is complete, the project manager then reviews the uploaded vaccination data on maps in the web platform to decide where to direct teams to next. NB Due to the copyright of Google Maps used as basemaps in the Mission Rabies App and web platforms, maps have been recreated in QGIS for this illustration. Displayed boundaries were created by Mission Rabies during project planning.

In addition to transfer of data from the field to the project manager, the system also enabled the team manager to effectively communicate the geographical boundaries within which the team was required to operate. Regions were manually created in GIS software using existing administrative boundaries, land type and dog population data to divide a region of work into contiguous zones of an appropriate size for a team to work in for approximately one day. The size of regions depended on the amount of data available for each project site; for example in Goa, regions containing approximately 100 dogs or of a size of 1km^2^ was applied. These contiguous zones were converted into KML format for uploading into the web platform where the project manager assigned each team regions using different colours, which appeared as boundaries on Google Maps within the smartphone forms (Fig 3). Teams then navigated with the help of their current location and a line showing the path of where they walked during that vaccination session (Path Tracker), with the aim of visiting all populated parts of the region and avoiding repeating areas that have already been completed. Toggling between Google Satellite and Google Road maps is of particular use in rural areas, enabling the user to visit all households during door-to-door work, even where defined roads do not exist.

A set of regions were generally allocated to each team for them to work through over several days, with the project manager reviewing progress on a daily basis on backend maps (Fig 3) and allocating additional regions as and when required. The exact number of regions and frequency of redirection varied by location, with the system having the flexibility for project managers to use an approach that suited their local project structure.

## Data review

Mapping functionality within the web-interface allowed the project managers to review team activity by plotting dog vaccination locations, static point clinic locations, dog sightings, schools visited and suspected rabies case locations by user on Google Maps (Fig 3). Path trackers were also reviewable in the web-platform to see the route walked by teams each day. Review of form data was performed by downloading datasets filtered by date and project in CSV format. These were then analysed with Microsoft Excel to generate summaries of vaccinations per team by different variables. More comprehensive analysis of project data were performed in R statistical software environment (https://cran.r-project.org) and QGIS Desktop (QGIS development team, Open Source Geospatial Foundation Project).

**Fig 4:**
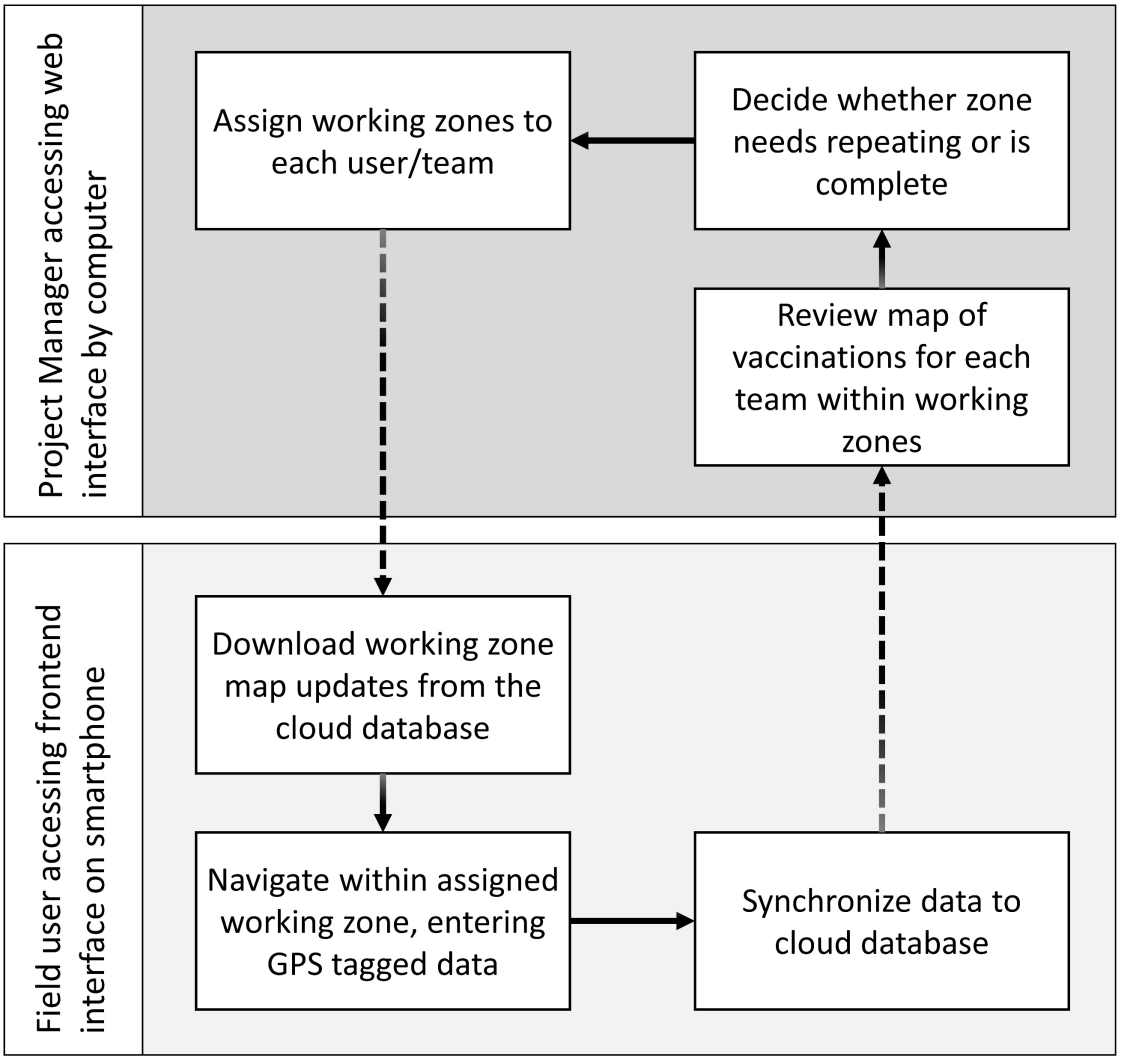
Flow diagram showing flow of data through the web and smartphone systems.

Fig 4 illustrates the flow of data from project manager through the web-based platform, to field staff using the Mission Rabies smartphone app. The geographic direction of teams based on review of the previous day or week’s work applied not only to vaccination teams, but also education, survey and rabies response activities. The app was used in Goa and Malawi to direct education teams ahead of vaccination teams, to increase awareness through classes in schools and meetings with local authorities, religious groups and community groups before vaccination activities ensued.

## Feedback

In addition to the iterative process of data submission, review and direction, project managers had a number of mechanisms to feedback information to users. These included a news section through which the project manager could broadcast summary reports and images to the project, and a chat forum through which users on the project could discuss logistical challenges such as stock shortages or technical issues.

## Results

### Data entry

**Fig 5:**
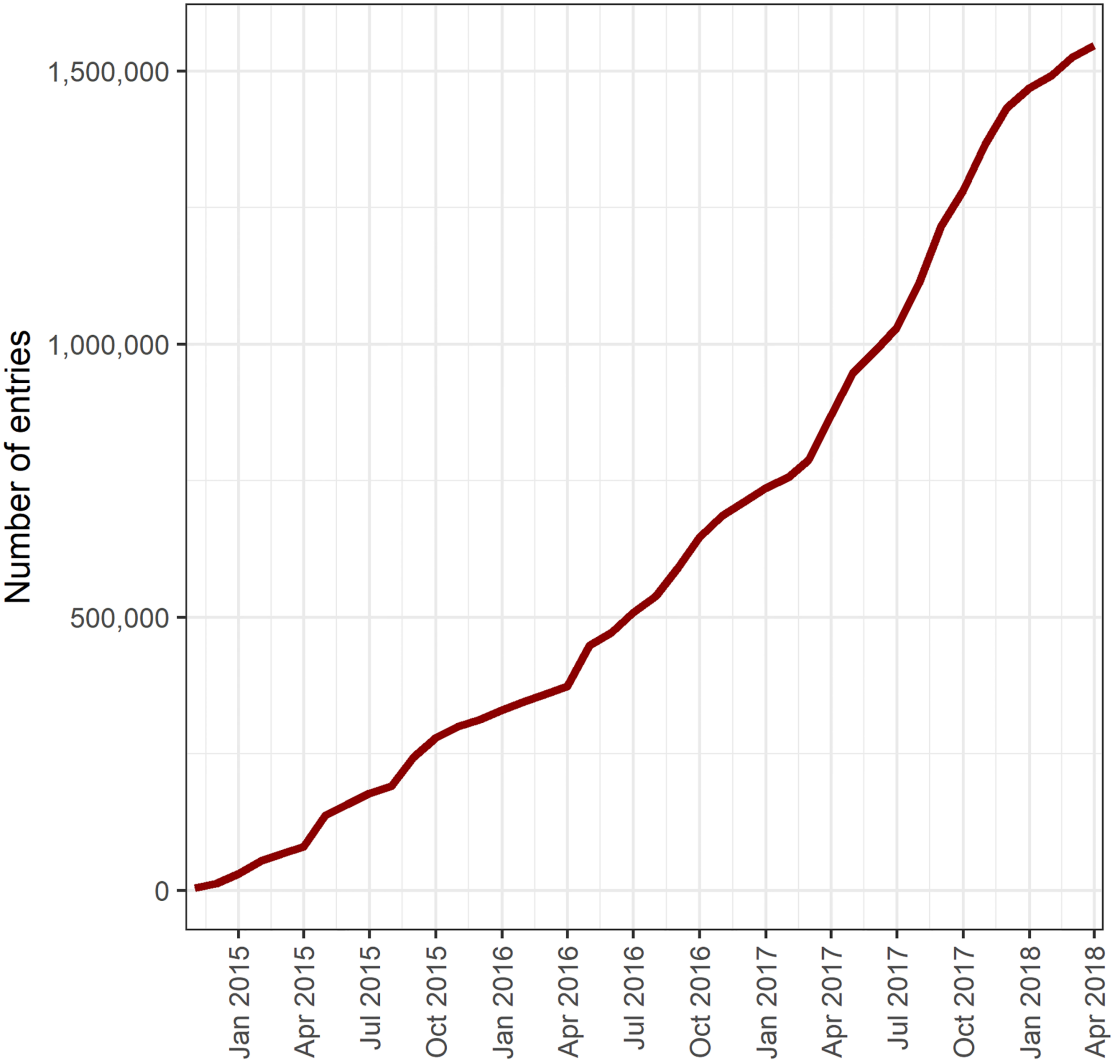
Cumulative frequency of data entries from 01/11/2014 to 22/04/2018.

**Fig 6:**
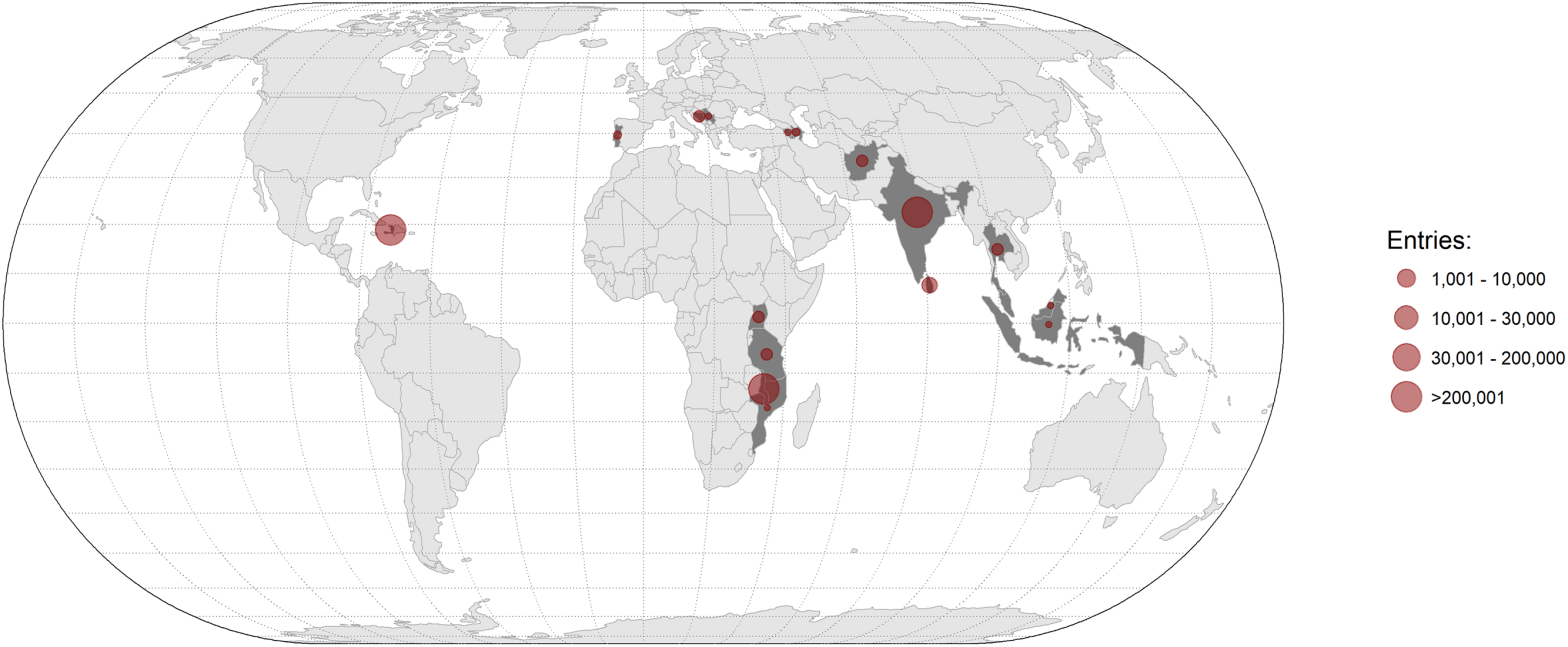
Map showing total number of app entries by country. Created in R Studio using ggplot. Country boundaries are openly available from http://gadm.org.

In total, 1,547,501 form entries were made on the app between 01/11/2014 to 22/04/2018 under categories of vaccination, surgery, survey and education (Table 1, Figs 5 and 6). The rate of data entry increased from 6,080 entries per month in 2014 to 60,195 per month in 2017 (S1 Fig).

**Table 1:** Table of summarised data by country showing the number of Mission Rabies App entries by category and the total number of vaccinations, education classes and children taught for the period 01/11/2014 to 22/04/2018.

	App Entries					Activity Summary		
Country	Vaccination Forms	Surgery Forms	Survey Forms	Education Forms	Total Entries	Total dogs vaccinated	School visits	Children taught
**India**	380,504	71,308	345,617	4,610	**802,039**	427,104	3,171	706,247
**Malawi**	319,605	2,398	40,327	3,175	**365,505**	267,036	847	642,304
**Haiti**	212,212		2,199		**214,411**	212,210		
**United Republic of Tanzania**	27,219	1,184	934		**29,337**	21,199		
**Uganda**	22,501	238	1,925		**24,664**	16,887		
**Sri Lanka**	21,703	16,779	26,905		**65,387**	34,326		
**Bosnia and Herzegovina**	9,050		9,303		**18,353**	9,015		
**Afghanistan**	7,469		3,911		**11,380**	7,459		
**Thailand**	1,841	7,717	2,409		**11,967**	9,553		
**Indonesia**	218				**218**	218		
**Armenia**		82	492		**574**	82		
**Azerbaijan**			1,068		**1,068**			
**Malaysia**			97		**97**			
**Portugal**			2,298		**2,298**			
**Serbia**		203			**203**	203		
**Total**	**1,002,322**	**99,909**	**437,485**	**7,785**	**1,547,501**	**1,005,292**	**4,018**	**1,348,551**

*Vaccination Form entries included vaccinations given to all species and records of already vaccinated dogs in some projects

** Survey Form entries included the dogs sighted during post vaccination surveys, population surveys and household surveys

In total, 1,005,292 dog vaccinations were recorded on the system, including 905,383 dogs vaccinated during field mass vaccination campaigns (90% of Vaccination Form entries) and a further 99,909 undergoing surgery (Table 1, S2 Fig). In many project sites, information about dogs found to already be vaccinated and other species vaccinated during the campaign were also recorded in Vaccination Forms, enabling campaign data to also provide wider insights into dog ownership and overall vaccine usage. The total number of children recorded in the app as being taught in school about rabies in project sites in India and Malawi during the study period was 1.3 million at 4,018 schools (Table 1).

### Team direction

Working zones were used on Mission Rabies project sites to direct vaccination teams in 12 areas across six countries (Table 2). The working zones were of a size that could approximately be covered by a vaccination team over a period of 1–3 days, making them a suitable unit to direct teams by, moving every few days. Furthermore these areas could generally be surveyed by two wheeler moped within a day, enabling the same regions to be used to direct surveyors conducting post vaccination assessments.

**Table 2:** Table of the number of working zones and total area covered for direction of teams on Mission Rabies vaccination projects.

Country	Region	Working zones	Area (km^2^)
India	Goa	425	3,684.2
	Ranchi	55	194.1
	Ooty	22	36.6
Malawi	Blantyre City	204	234.5
	Blantyre rural	131	883.8
	Chiradzulu	106	544.6
	Zomba City	30	45.2
	Zomba rural	263	1397.0
Uganda	Koch Goma	10	315.7
Sri Lanka	Negombo	103	131.7
Tanzania	Meru	24	19.6
Thailand	Chiang Mai	6	21.8
**Total**		**1,379**	**7,508.8**

### Department coordination

**Fig 7:**
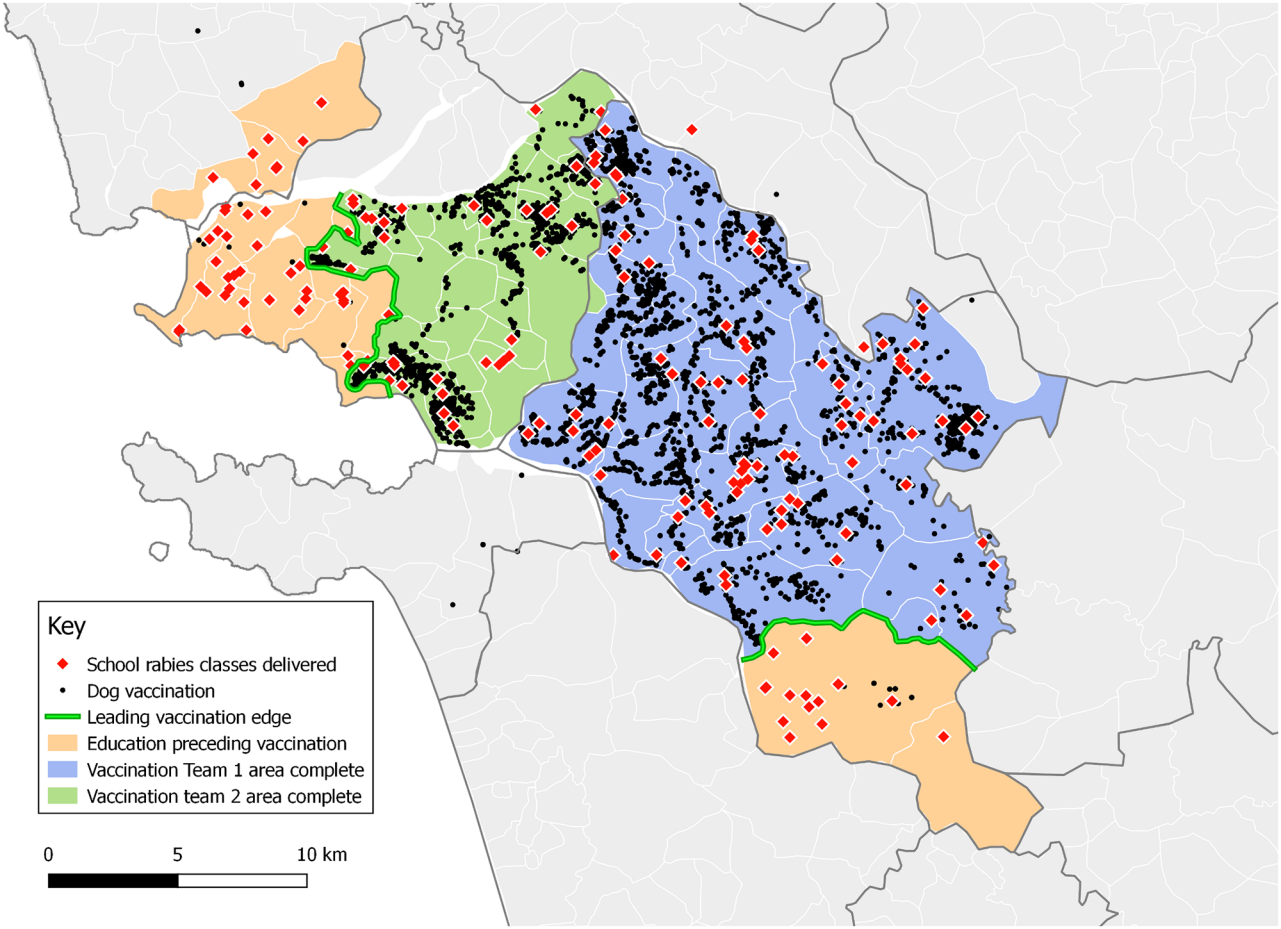
Map showing the movement of two groups of education teams ahead of two vaccination teams in Goa from March to June 2017. Created in QGIS using data from the Mission Rabies App. Goa administrative boundaries were recreated by Mission Rabies from government administrative maps.

The app was used to support the coordination of vaccination and education departments in Goa through direction to specific map regions. Fig 7 illustrates a period of work in Goa from March to June 2017, where regions are targeted by education teams in the period before vaccination begins. During this period, rabies education teams delivered classes to 24,927 children, representing 89% of students at the schools visited. Vaccination teams followed to vaccinate 10,131 dogs through the same regions.

### Development and scalability

During the period of study, the app was improved and refined, with addition of new functionalities in response to user feedback. These were released to users through updates which needed to be installed periodically. In November 2016 the system was updated to facilitate the creation of multiple projects, grouped under a single organisation. This enhanced data security and scalability of the system to multiple independent organisations. In March 2017 the addition of a simple “counter” functionality was created for the Haiti setting, enabling the rapid capture of the date, time and GPS for each dog vaccinated through the click of a button. Enhancement of the backend mapping functions and region allocation of individual users has been an ongoing area of development to improve the usability and data review for project managers.

**Fig 8:**
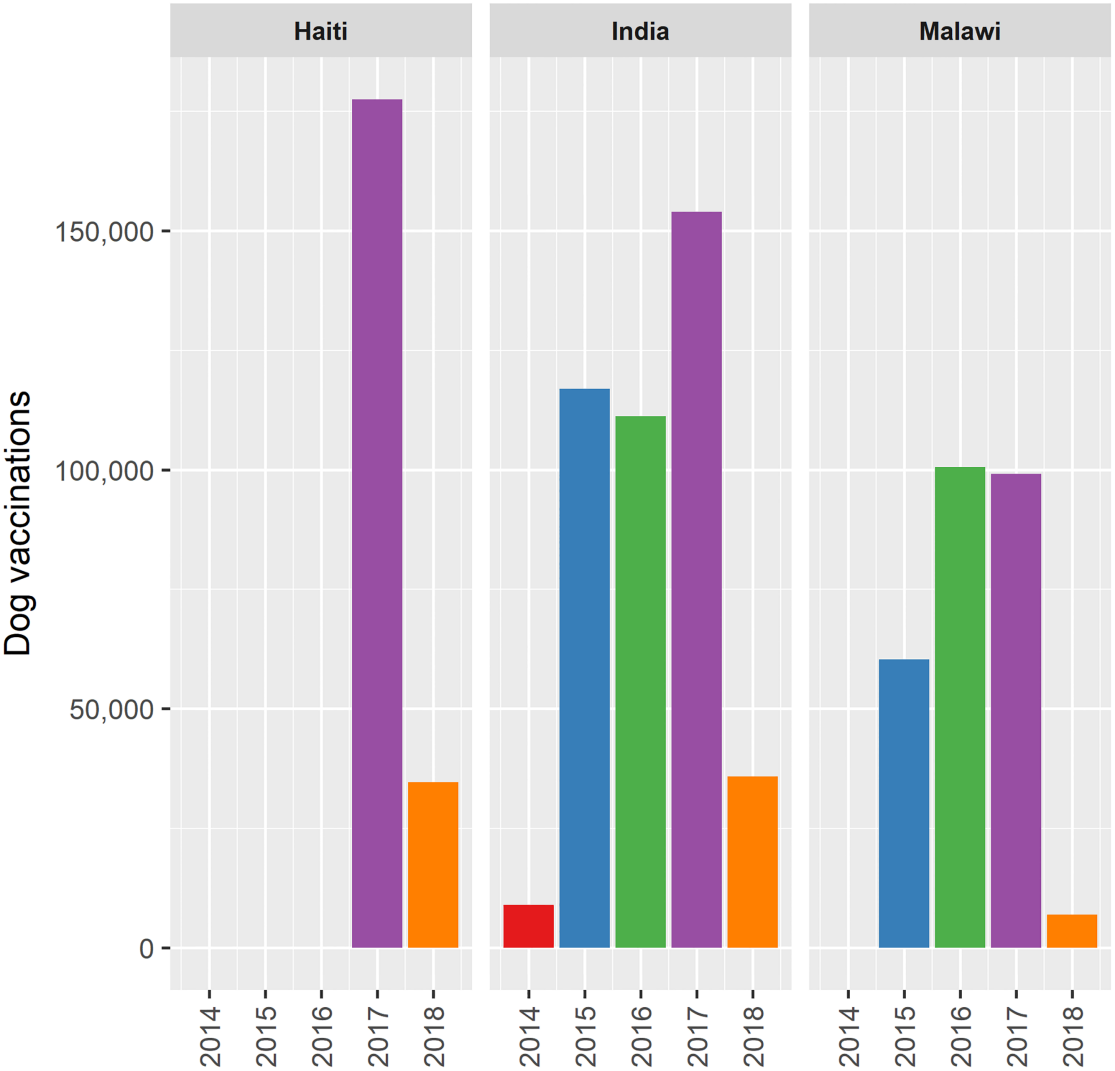
Bar graph of annual number of entries recorded on the Mission Rabies App in Haiti, India and Malawi by year.

The progressive increase in usage of the system has coincided with expansion of the Goa campaign from a focal pilot project in 2014 to a systematic campaign covering the entire State of Goa (Fig 8). In 2017 the Centres for Disease Control and Prevention used the app to support the national mass dog vaccination campaign in Haiti, implemented by the Ministry of Agriculture (MARNDR). Feedback on the app from the Chief Veterinary Officer of Haiti included “The Mission Rabies [App] maps that show the day’s vaccination record are very helpful to me as the CVO when I want to update… the Minister of Agriculture of Haiti on the progress of our rabies vaccination campaign.” (M Millien, personal communication March 2018).

## Discussion

There is growing emphasis on the global effort to eliminate dog-mediated human rabies by 2030 [5], however challenges remain in coordinating large scale, effective mass dog vaccination campaigns in many endemic regions. Here we describe a unique mHealth innovation which was successfully used at scale across project sites in numerous countries to enhance the remote oversight and direction of dog vaccination teams recording the details of over one million dog vaccinations. This is a rare example of an mHealth system which has been successfully scaled to national and India state level implementation. It demonstrates the potential for transfer of functionalities across several rabies endemic settings, enhancing data capture and reporting in mass dog vaccination campaigns as momentum builds towards the global 2030 goal of canine transmitted human rabies elimination.

Innovations in mHealth make it possible to monitor and evaluate activities of remote workers, yet these benefits have not been previously demonstrated in large scale rabies control interventions. Examples where mHealth has benefitted mass vaccination activities include mass vaccination of people against cholera in Haiti [18] and in the eradication of polio in low income countries [17], however there are few examples where such initiatives have been established in sustained expanding campaigns [22]. The outcome of focal mass vaccination campaigns using the Mission Rabies App has been previously reported [19,20], however this is the first description of the use of smartphones to direct mass vaccination activities across multiple settings and at this scale.

The novel flow of information from project managers back to field staff, based on their assessment of the inflowing data has not been previously reported in field based mHealth data capture systems [13,17,18,23,24]. This two-way data flow enabled for a dynamic project structure where the project manager could adapt their vaccination plan, based on team progress. This required project managers to become familiar with the backend system, however the simple interface makes it possible to map GPS vaccination points on Google Maps through the simple selection of dates within a given project. Using this approach did, however, require the managers to have regular access to reliable internet connection during the campaign to view data on the backend and assign new regions, as well as having the time to login and review team progress. A comprehensive comparison study has not yet been performed, however, the successful adoption of the system at scale across numerous project locations indicates that the system is usable through a range of project management structures. In several project sites, the assignment of multiple regions for teams to sequentially work through over several days made it possible to reduce the frequency of data review by the project manager from daily to once or twice weekly. The use of date-scheduled region assignment has been considered to further reduce the need for project managers to review data, however this has been hampered by the changing pace of work which requires regular tweaking of the schedule and for the project manager to keep up to date with team movements. Self-direction of individual teams is also something to explore, however we anticipate that an element of review and direction will always be needed from an authority.

Technology makes it possible to automate many activities that there otherwise is not the capacity or expertise available to complete. In this example, many campaigns would not have the time to collate and report on hundreds of paper reports from vaccination teams, whilst the system automatically gathers and presents these data in near real-time. Similarly, it is not feasible to train all project coordinators in the use of mapping (GIS) software, however field data can be presented on maps through the simple selection of users and dates. This level of monitoring and review would simply not be possible through paper-based data records [23,25]. Further investigation to estimate operational impact on campaign efficiency as a result of the system is currently underway within specific project sites.

A focus on intuitive design made it possible for existing staff to begin using the system to enter data with minimal training. Further study is needed to assess any impact on team-level working practices resulting from the enhanced monitoring gained through the app, however other studies have reported improved engagement and motivation where staff know their efforts will be reviewed and acknowledged as a result of recording through technology [16,24]. The same may be true for a worker sent to vaccinate every part of a village in that they may be more motivated to visit every street if they know their path is being recorded and visible to their manager.

The system was customisable enough for individual project sites to use it in a way that worked within their campaign structure. In Mission Rabies campaigns, stakeholders such as the Government of Goa and Dogs Trust were supportive of gathering data relating to ownership, dog health and reproduction. The recording of these data at the individual dog level during the campaign made it possible to monitor the proportion of stray dogs, ownership practices (e.g. confinement) and welfare indicators (e.g. incidence of skin disease) over sequential campaigns. To avoid slowing the rate of vaccination it was necessary to employ an additional member of staff in each team to record information about every dog. In Haiti, however, indicators of interest in the dog population were monitored through periodic surveys at key time points, and so investing time in detailed data capture of every dog during the campaign was not a priority. The benefit of the app was in being able to direct teams to specific regions and record key operational outputs such as the date, time and location of every vaccine administered by each user. This was done through the simple click of a button for each vaccination and so could be done within the existing project infrastructure with minimal impact on vaccination capacity. Further study is underway to quantify this finding.

The ability to capture and store data offline negated the need for network coverage in the region of work, however an internet connection was required to synchronize data to the server and therefore share information with the supervision team and to refresh regions for team direction. There was usually an opportunity to connect once a day or every few days for this purpose and it is expected that this will continue to improve as local network infrastructures expand [26]. In areas where regular connection is not available for extended periods of the campaign, all regions can be assigned at the start of the campaign for teams to work through sequentially offline and uploading all data at the end of the campaign. In this case adjustment to the approach would need to be done, either through local review of vaccination activity on the phones or at the end of the campaign. Limitations of battery life and the need for charging points were overcome using power banks for use in the field or adaptors for charging from vehicles.

Coordination of activities that engage communities in rabies control have been shown to enhance the efficacy of mass vaccination campaigns [27–29]. In this example the app has been used to ensure community/school education teams immediately preceded mass vaccination of a geographic area. This is the first example that we know of that has used mobile technology to coordinate public education teams ahead of vaccination activities in this way.

The tools to eliminate rabies are already available [6], however the remaining obstacles in progressing towards rabies elimination are political, financial and operational [30–33]. Novel funding structures such as Development Impact Bonds have the potential to generate new investment, however these require robust evaluation of outputs [33–35]. Individual digital recording of each vaccination may be of benefit in verifying campaign outputs as a component of such funding structures. Aggregation of individual dog data at the ward, municipality, district, state and national levels provides a new level of transparency and oversight at every tier of management in massive campaigns. The depth and validity of reporting that then becomes available may be of benefit in motivating government officials to sustain and expand such activities as well as in reporting on funding such as Development Impact Bonds.

## Conclusion

WHO and global experts on rabies emphasise the urgent need for innovation to support the rapid up-scaling of rabies control activities such as mass dog vaccination and educating communities about correct post-exposure prophylaxis. Mobile health (mHealth) tools are increasing the ability to send and receive data from remote public health interventions, improving campaign reporting and coordination, however there are few examples where this has been successfully applied at a national scale. Here we describe a novel innovation using smartphone technology to facilitate the management and monitoring of projects undertaking systematic vaccination and education across large geographic areas. Such systems have the potential to provide the monitoring and evaluation structure, as well as remote management, to enable the expansion of mass vaccination efforts to the regional, national and international level, which is essential in progressing towards rabies elimination. These methods could also be applied to other public health interventions which require the remote monitoring and geographic direction of staff for the purpose of vaccination, animal and human health monitoring and population surveys. The methods presented here provide the foundation for further investigations to evaluate the impact of this technology on operational output, project efficiencies and cost-benefit.

## Supporting information

S1 Fig Mean monthly entries on the Mission Rabies App by year. NB mean entries per month in 2018 is currently low due to the majority of 2018 campaigns beginning after the period of study.
(PNG)

S2 Fig Number of entries on the Mission Rabies App by category, year and country. (PNG)

S1 Table Table of common data entry fields included in customised forms for different categories of work. (PDF)

## References

[1] Cleaveland S, Kaare M, Tiringa P, Mlengeya T, Barrat J. A dog rabies vaccination campaign in rural Africa: impact on the incidence of dog rabies and human dog-bite injuries. Vaccine. 2003;21: 1965–1973. 10.1016/S0264-410X(02)00778-8 12706685

[2] Belotto A, Leanes LF, Schneider MC, Tamayo H, Correa E. Overview of rabies in the Americas. Virus Res. 2005;111: 5–12. 10.1016/j.virusres.2005.03.006 15896398

[3] Schneider MC, Belotto A, Adé MP, Hendrickx S, Leanes LF, Rodrigues MJDF, et al Current status of human rabies transmitted by dogs in Latin America. Cad Saude Publica. 2007;23: 2049–2063. 10.1590/S0102-311X2007000900013 17700940

[4] Hampson K, Coudeville L, Lembo T, Sambo M, Kieffer A, Attlan M, et al Estimating the Global Burden of Endemic Canine Rabies. PLoS Negl Trop Dis. 2015;9 10.1371/journal.pntd.0003709 25881058 PMC4400070

[5] Wallace RM, Undurraga EA, Blanton JD, Cleaton J, Franka R. Elimination of Dog-mediated human rabies deaths by 2030: Needs assessmnet and alternatives for progress based on dog vaccination. Front Vet Sci. 2017;4: 1–14. 28239608 10.3389/fvets.2017.00009 PMC5300989

[6] Lembo T, Hampson K, Kaare MT, Ernest E, Knobel D, Kazwala RR, et al The feasibility of canine rabies elimination in Africa: dispelling doubts with data. PLoS Negl Trop Dis. 2010;4: e626 10.1371/journal.pntd.0000626 20186330 PMC2826407

[7] Jibat T, Hogeveen H, Mourits MCM. Review on Dog Rabies Vaccination Coverage in Africa: A Question of Dog Accessibility or Cost Recovery? PLoS Negl Trop Dis. 2015;9: e0003447 10.1371/journal.pntd.0003447 25646774 PMC4315526

[8] Ferguson EA, Hampson K, Cleaveland S, Consunji R, Deray R, Friar J, et al Heterogeneity in the spread and control of infectious disease: consequences for the elimination of canine rabies. Sci Rep. Nature Publishing Group; 2015;5: 18232 10.1038/srep18232 26667267 PMC4678884

[9] Hampson K, Dushoff J, Bingham J, Brückner G, Ali YH, Dobson A. Synchronous cycles of domestic dog rabies in sub-Saharan Africa and the impact of control efforts. Proc Natl Acad Sci U S A. 2007;104: 7717–22. 10.1073/pnas.0609122104 17452645 PMC1863501

[10] De Carvalho MF, Vigilato MAN, Pompei JA, Rocha F, Vokaty A, Flores BM, et al Rabies in the Americas: 1998–2014. PLoS Negl Trop Dis. 2018;12: e0006271 10.1371/journal.pntd.0006271 29558465 PMC5877887

[11] Ali M, Park J-K, von Seidlein L, Acosta CJ, Deen JL, Clemens JD. Organizational aspects and implementation of data systems in large-scale epidemiological studies in less developed countries. BMC Public Health. 2006;6: 86 10.1186/1471-2458-6-86 16584571 PMC1450272

[12] Bernabe-Ortiz A, Curioso WH, Gonzales MA, Evangelista W, Castagnetto JM, Carcamo CP, et al Handheld computers for self-administered sensitive data collection: a comparative study in Peru. BMC Med Inform Decis Mak. 2008;8: 11 10.1186/1472-6947-8-11 18366687 PMC2323371

[13] Aanensen DM, Huntley DM, Feil EJ, al-Own F, Spratt BG. EpiCollect: linking smartphones to web applications for epidemiology, ecology and community data collection. PLoS One. 2009;4: e6968 10.1371/journal.pone.0006968 19756138 PMC2735776

[14] Thriemer K, Ley B, Ame SM, Puri MK, Hashim R, Chang NY, et al Replacing paper data collection forms with electronic data entry in the field: findings from a study of community-acquired bloodstream infections in Pemba, Zanzibar. BMC Res Notes. 2012;5: 113 10.1186/1756-0500-5-113 22353420 PMC3392743

[15] Avilés W, Ortega O, Kuan G, Coloma J, Harris E. Quantitative assessment of the benefits of specific information technologies applied to clinical studies in developing countries. Am J Trop Med Hyg. 2008;78: 311–5. 18256435

[16] Mtema Z, Changalucha J, Cleaveland S, Elias M, Ferguson M, Halliday JEB, et al Mobile Phones As Surveillance Tools: Implementing and Evaluating a Large-Scale Intersectoral Surveillance System for Rabies in Tanzania. PLoS Negl Trop Dis. 2016; 1–12. 10.1371/journal.pmed.1002002 27070315 PMC4829224

[17] Kim SS, Patel M, Hinman A. Use of m-Health in polio eradication and other immunization activities in developing countries. Vaccine. Elsevier Ltd; 2017;35: 1373–1379. 10.1016/j.vaccine.2017.01.058 28190744

[18] Teng JE, Thomson DR, Lascher JS, Raymond M, Ivers LC. Using Mobile Health (mHealth) and Geospatial Mapping Technology in a Mass Campaign for Reactive Oral Cholera Vaccination in Rural Haiti. PLoS Negl Trop Dis. 2014;8 10.1371/journal.pntd.0003050 25078790 PMC4117440

[19] Gibson AD, Ohal P, Shervell K, Handel IG, Bronsvoort BM, Mellanby RJ, et al Vaccinate-assess-move method of mass canine rabies vaccination utilising mobile technology data collection in Ranchi, India. BMC Infect Dis. BMC Infectious Diseases; 2015;15: 589 10.1186/s12879-015-1320-2 26715371 PMC4696259

[20] Gibson AD, Handel IG, Shervell K, Roux T, Mayer D, Muyila S, et al The Vaccination of 35,000 Dogs in 20 Working Days Using Combined Static Point and Door-to-Door Methods in Blantyre, Malawi. PLoS Negl Trop Dis. 2016;10: e0004824 10.1371/journal.pntd.0004824 27414810 PMC4945057

[21] Burdon Bailey JL, Gamble L, Gibson AD, Bronsvoort BMdC, Handel IG, Mellanby RJ, et al A rabies lesson improves rabies knowledge amongst primary school children in Zomba, Malawi. PLoS Negl Trop Dis. 2018;12: 1–16. 10.1371/journal.pntd.0006293 29522517 PMC5862537

[22] Malanga DF, Chigona W. Mobile Health Initiatives in Malawi: Understanding Impact, Funding. Int J Priv Heal Inf Manag. 2018; 10.4018/IJPHIM.2018010104

[23] Sherin S, Mathew P, Johns F, Abraham J. The feasibility of using remote data collection tools in field surveys. Int J Community Med Public Heal. 2018;5: 81–85.

[24] Stanton M, Molineux A, Mackenzie C, Kelly-Hope L. Mobile Technology for Empowering Health Workers in Underserved Communities: New Approaches to Facilitate the Elimination of Neglected Tropical Diseases. JMIR Public Heal Surveill. 2016;2: e2 10.2196/publichealth.5064 27227155 PMC4869228

[25] Mwabukusi M, Karimuribo ED, Rweyemamu MM, Beda E. Mobile technologies for disease surveillance in humans and animals. Onderstepoort J Vet Res. 2014;81: 1–5. 10.4102/ojvr.v81i2.737 25005126

[26] International Telecommunication Union (ITU). ITC Facts and 2017 figures [Internet]. 2017 [cited 6 May 2018]. https://www.itu.int/en/ITU-D/Statistics/Pages/facts/default.aspx

[27] Widyastuti MDW, Bardosh KL, Sunandar, Basri C, Basuno E, Jatikusumah A, et al On dogs, people, and a rabies epidemic: results from a sociocultural study in Bali, Indonesia. Infect Dis Poverty. Infectious Diseases of Poverty; 2015;4: 30 10.1186/s40249-015-0061-1 26137295 PMC4486702

[28] Kaare M, Lembo T, Hampson K, Ernest E, Estes A, Mentzel C, et al Rabies control in rural Africa: evaluating strategies for effective domestic dog vaccination. Vaccine. 2009;27: 152–160. 10.1016/j.vaccine.2008.09.054 18848595 PMC3272409

[29] Mazeri S, Gibson AD, Meunier N, Bronsvoort BMdC, Handel IG, Mellanby RJ, et al Barriers of attendance to dog rabies static point vaccination clinics in Blantyre, Malawi. PLoS Negl Trop Dis. 2018;12 10.1371/journal.pntd.0006159 29324737 PMC5783422

[30] Bourhy H, Dautry-Varsat A, Hotez PJ, Salomon J. Rabies, still neglected after 125 years of vaccination. PLoS Negl Trop Dis. 2010;4: e839 10.1371/journal.pntd.0000839 21152052 PMC2994912

[31] Cleaveland S, Hampson K, Cleaveland S. Rabies elimination research: juxtaposing optimism, pragmatism and realism. Proc R Soc B Biol Sci. 2017;284 10.1098/rspb.2017.1880 29263285 PMC5745407

[32] Tohma K, Saito M, Demetria CS, Manalo DL, Quiambao BP, Kamigaki T, et al Molecular and mathematical modeling analyses of inter-island transmission of rabies into a previously rabies-free island in the Philippines. Infect Genet Evol. 2016;38: 22–28. 10.1016/j.meegid.2015.12.001 26656835

[33] Welburn S, Coleman C, Zinsstag J. Rabies Control: Could innovative Financing Break the Deadlock? Front Vet Sci. 2017;4: 1–8. 28337440 10.3389/fvets.2017.00032 PMC5343007

[34] Cleaveland S, Lankester F, Townsend S, Lembo T, Hampson K. Rabies control and elimination: a test case for One Health. Vet Rec. 2014;175: 188–93. 10.1136/vr.g4996 25172649 PMC7612423

[35] Welburn SC, Bardosh KL, Coleman PG. Novel Financing Model for Neglected Tropical Diseases: Development Impact Bonds Applied to Sleeping Sickness and Rabies Control. PLoS Negl Trop Dis. 2016;10: 1–5. 10.1371/journal.pntd.0005000 27855156 PMC5113866

