# Supplementary figures and images for: One million dog vaccinations recorded on mHealth innovation used to direct teams in numerous rabies control campaigns
Source: PLoS One. 2018 Jul 26;13(7):e0200942. doi: 10.1371/journal.pone.0200942 (PMC6062050; doi:10.1371/journal.pone.0200942)

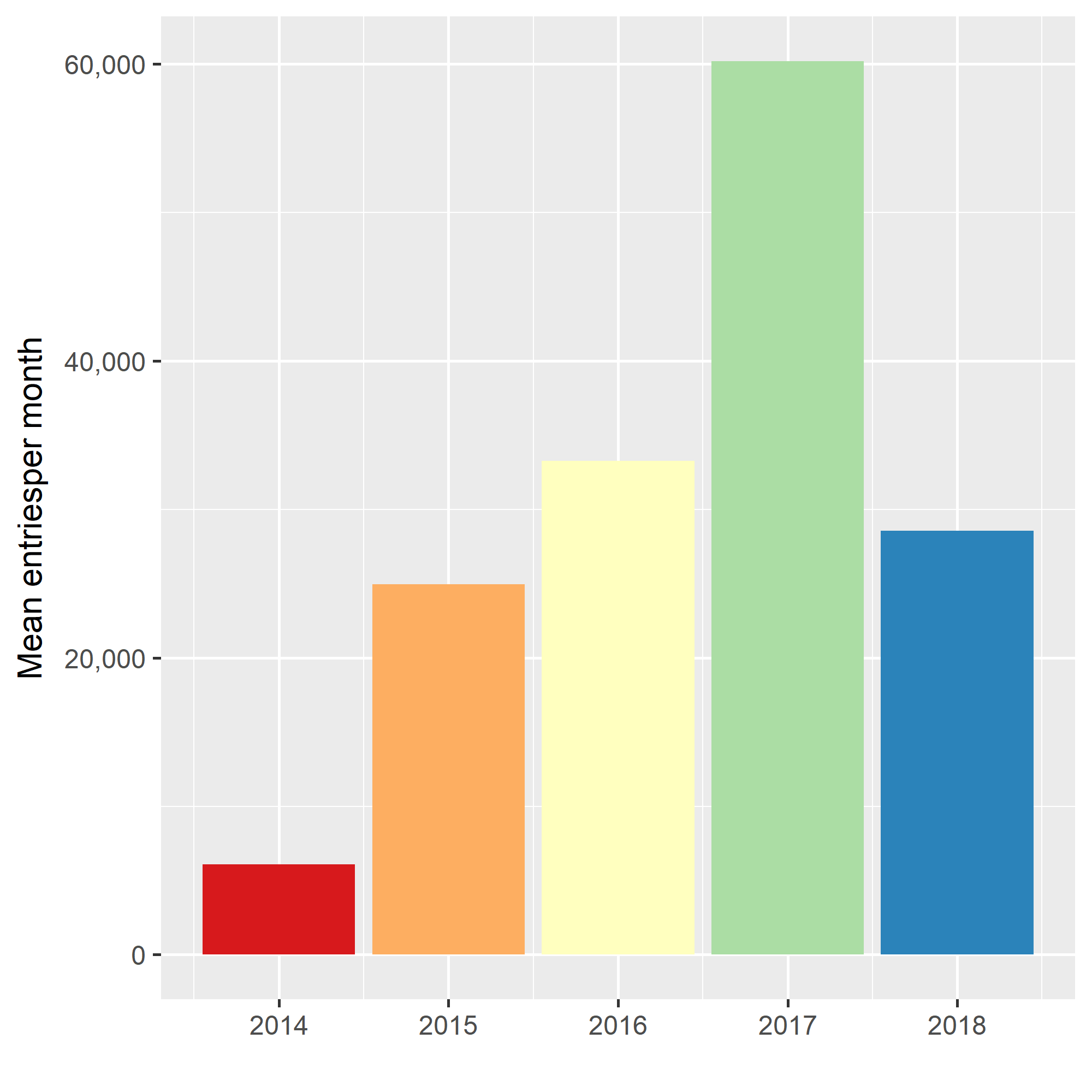

Supplement: S1 Fig — NB mean entries per month in 2018 is currently low due to the majority of 2018 campaigns beginning after the period of study. (PNG) [file pone.0200942.s001.png]

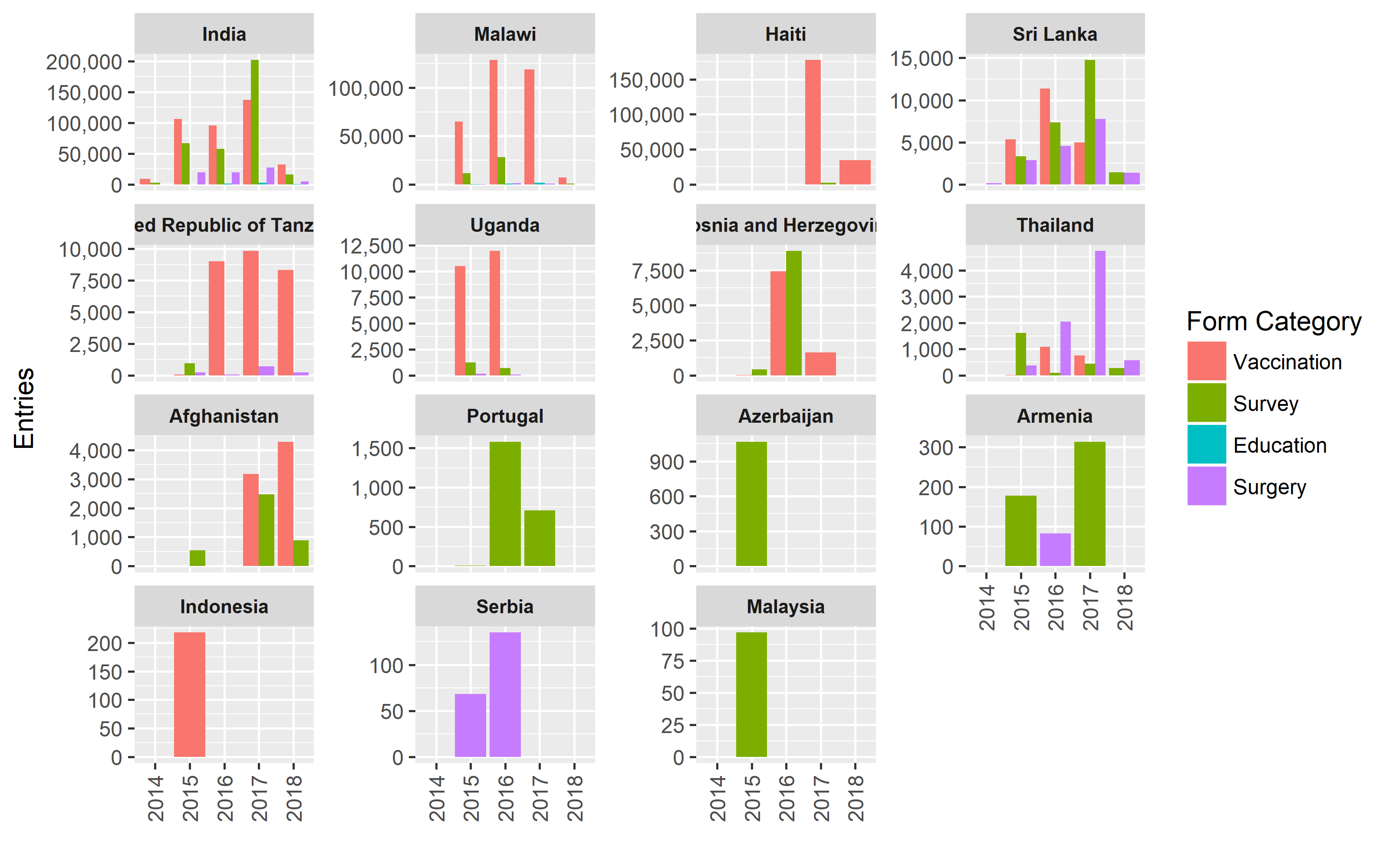

Supplement: S2 Fig — (PNG) [file pone.0200942.s002.png]
