# Supplementary material for: One million dog vaccinations recorded on mHealth innovation used to direct teams in numerous rabies control campaigns
Source: PLoS One. 2018 Jul 26;13(7):e0200942. doi: 10.1371/journal.pone.0200942 (PMC6062050; doi:10.1371/journal.pone.0200942)
Supplement: S1 Table — (PDF) [file pone.0200942.s003.pdf]

Supporting Information 1

SI 1: Table of common data entry fields included in customised forms for different categories of work

Vaccination Form	Post-Vaccination Survey Form	Education Record Form	Rabies Response Form
• Action<br>- Vaccinated<br>- Already vaccinated<br>- Not vaccinated<br>• Sex<br>- Male<br>- Female lactating<br>- Female non-lactating<br>- Unknown<br>• Age<br>- Adult (>3months)<br>- Puppy (<3months)<br>• Ownership<br>- Owned<br>- Stray<br>• Confinement<br>- Roaming<br>- Confined<br>• Neuter status<br>- Entire<br>- Neutered<br>- Unknown<br>• Health status<br>- Healthy<br>- Sick or injured<br>• Comments	• Vaccination status<br>- Mark present<br>- Mark absent<br>• Sex<br>- Adult male<br>- Adult non-lactating female<br>- Adult lactating female<br>- Puppy<br>- Unknown<br>• Neuter status<br>- Entire<br>- Neutered<br>- Unknown	• School name<br>• Type of school<br>- Primary<br>- Secondary<br>- High School<br>• Grades taught in school<br>• Total number of children in school<br>• Grades rabies educated<br>• Number of children rabies educated<br>• Class delivered<br>- Single classes<br>- Group classes<br>- Assembly<br>- Other (describe)<br>• Activities conducted<br>- Presentation<br>- Quiz<br>- Drama<br>• Comments	• History<br>• Clinical signs<br>• Ownership status<br>- Owned<br>- Stray<br>• Confinement<br>- Roaming<br>- Confined<br>• Number of people bitten<br>• Number/type of animals bitten<br>• Outcome<br>- Dog not caught<br>- Home quarantine<br>- Dog removed<br>- Dead on arrival
